# LC-MS-Based Global Metabolic Profiles of Alternative Blood Specimens Collected by Microsampling

**DOI:** 10.3390/metabo15010062

**Published:** 2025-01-16

**Authors:** Marlene N. Thaitumu, Daniel Marques De Sá e Silva, Philippine Louail, Johannes Rainer, Glykeria Avgerinou, Anatoli Petridou, Vassilis Mougios, Georgios Theodoridis, Helen Gika

**Affiliations:** 1 Department of Medicine, Aristotle University of Thessaloniki, 54124 Thessaloniki, Greece; mthai@auth.gr; 2 Biomic AUTh, Center for Interdisciplinary Research and Innovation (CIRI-AUTH), Balkan Center, B1.4, 57001 Thessaloniki, Greece; dmarque@auth.gr (D.M.D.S.e.S.); gtheodor@chem.auth.gr (G.T.); 3 Department of Chemistry, Aristotle University of Thessaloniki, 54124 Thessaloniki, Greece; 4 Institute for Biomedicine, Eurac Research, 39100 Bolzano, Italy; philippine.louail@eurac.edu (P.L.); johannes.rainer@eurac.edu (J.R.); 5 School of Physical Education & Sport Science at Thessaloniki, Aristotle University of Thessaloniki, 57001 Thessaloniki, Greece; gavgeri@phed.auth.gr (G.A.); apet@phed.auth.gr (A.P.); mougios@auth.gr (V.M.)

**Keywords:** blood microsampling (BµS), dried blood spot (DBS), quantitative dried blood spots (qDBS), volumetric absorptive microsampling (VAM), liquid chromatography–mass spectrometry (LC-MS), metabolome, blood metabolites, global metabolic profile, untargeted metabolomics, blood metabolic phenotype

## Abstract

Blood microsampling (BμS) has recently emerged as an interesting approach in the analysis of endogenous metabolites but also in metabolomics applications. Their non-invasive way of use and the simplified logistics that they offer renders these technologies highly attractive in large-scale studies, especially the novel quantitative microsampling approaches such as VAMs or qDBS. **Objectives:** Herein, we investigate the potential of BµS devices compared to the conventional plasma samples used in global untargeted mass spectrometry-based metabolomics of blood. **Methods:** Two novel quantitative devices, namely, Mitra, Capitainer, and the widely used Whatman cards, were selected for comparison with plasma. Venous blood was collected from 10 healthy, overnight-fasted individuals and loaded on the devices; plasma was also collected from the same venous blood. An extraction solvent optimization study was first performed on the three devices before the main study, which compared the global metabolic profiles of the four extracts (three BµS devices and plasma). Analysis was conducted using reverse phase LC-TOF MS in positive mode. **Results:** BµS devices, especially Mitra and Capitainer, provided equal or even superior information on the metabolic profiling of human blood based on the number and intensity of features and the precision and stability of some annotated metabolites compared to plasma. Despite their rich metabolic profiles, BµS did not capture metabolites associated with biological differentiation of sexes. **Conclusions:** Overall, our results suggest that a more in-depth investigation of the acquired information is needed for each specific application, as a metabolite-dependent trend was obvious.

## 1. Introduction

Metabolomics, the comprehensive, unbiased profiling of the metabolic content in a biological sample, is the fastest-growing “omics” technology with great potential in clinical diagnostics and population health monitoring and prevention [1,2,3]. Blood metabolome read-outs, providing insights into the physiology of the organism under study, hold great promise in improving risk prediction and diagnosis and enable more precise prognosis of disease progression, addressing important clinical needs, and facilitating the shift in healthcare from a proactive to a predictive, preventive, personalized, and participatory (P4) medicinal approach [4,5,6,7].

Metabolomics research for these purposes involves large-scale studies, multi-center sample collection, or longitudinal follow-up of subjects, which often generate limitations associated with sample collection, handling, and stability. Blood microsampling (BµS) approaches offer significant advantages to mitigate such problems, rendering them a highly attractive approach in blood metabolomics research. Ease and higher compliance from venipuncture-averse individuals (newborns, children, and the elderly), lower costs due to the self-collection potential, and simplified logistics are some of the features that can enhance their widespread application in metabolomics [8,9,10,11] and open new routes in disease biomarker discovery and/or biomarker detection for health monitoring.

Indeed, over the last few years, an increased interest has emerged in the use of several microsampling approaches in blood metabolomics, indicating such a perspective [12]. Nevertheless, in order to incorporate the use of such technologies in untargeted metabolomics, comprehensive studies are required to ensure that similarly useful information on circulating metabolites can be acquired compared to conventional blood matrices [13,14]. Thus, the application of BµS within metabolomics workflows still needs in-depth investigation and evaluation.

While there have been numerous publications on BµS versus conventional blood matrices in targeted metabolomics [15,16,17,18], to date, only a few have employed untargeted metabolomics [19,20,21,22,23] or lipidomics analysis [24,25,26,27,28,29,30]. Of those on metabolomics, three used Whatman cards [19,20,21], three used Mitra [21,22,23], and only one used both [21]. In most of these studies, LC-MS-based metabolic profiling was performed [19,20,22,23], while two studies used GC-MS either alone [21] or in combination with LC-MS [20]. Findings in dried venous blood were compared to that of plasma [19,21,23], whereas in two cases, dried venous blood was compared with both capillary blood and plasma [20,22].

These works aimed to either compare the metabolome obtained from the fingertip to those of plasma and venous blood in healthy individuals [22] or to exhibit the utility of BμS in the context of case–control metabolomics studies, such as in pregnant women with HIV [19] or in breast cancer patients [21]. The potential of BμS was also demonstrated in studies applying repetitive sampling from the same individuals, either examining the temporal metabolite-level fluctuations within hours and days of sample collection [20] or exploring lifestyle-associated changes in health [23].

While these studies provided information on the metabolic profiles acquired from BµS following untargeted approaches, further research and thorough examination are required for a clear understanding of the advantages offered by microsampling devices over plasma or serum that are typically used. None of the aforementioned studies report optimization experiments on sample extraction, which can critically affect the metabolomic profiles obtained, given that different BµS devices are manufactured with varied materials. Furthermore, these studies used only two types of BµS devices, Mitra and Whatman cards. As the availability of BµS devices in the market increases, comprehensive studies comparing multiple devices are necessary to provide insight into factors such as cost, ease of use, reproducibility, and metabolome capture. The present study aims to fill this gap by comparing three devices: Whatman cards, Capitainer B, and Mitra. First, optimization of the extraction procedures for the selected BµS devices to obtain the most comprehensive profile was performed. Then, comparing the metabolic profiles obtained from the BµS devices with that obtained from a conventional blood matrix, plasma was studied. To this end, venous blood was collected by venipuncture from 10 healthy individuals and applied to the BµS devices. Plasma from the same individuals was also obtained. Analysis was performed using a reverse-phase (RP) global untargeted metabolic profiling LC–quadrupole time-of-flight–MS (LC-qTOFMS) method. To the best of our knowledge, this is the first comprehensive study that compares global metabolic profiles from three different BµS devices, the first for the Capitainer B device over plasma through an untargeted LC-MS-based metabolomic approach.

## 2. Materials and Methods

### 2.1. Chemicals and Reagents

All solvents used were LC-MS grade. Acetonitrile (AcN), methanol (MeOH), and formic acid were acquired from VWR BDA Chemicals (Radnor, PA, USA). Sodium hydroxide, ^13^C-Phenylalanine (^13^C-Phe), butyrylcarnitine-d3 (BC-d3), and octanoylcarnintine-d3 (OC-d3) were purchased from Sigma-Aldrich (St. Louis, MI, USA). Isopropanol (IPA) was from Chem-Lab (Zedelgem, Belgium). Ultrapure water was produced using a Hydrolab demineralizer (Straszyn, Poland).

### 2.2. Microsampling Devices

Twenty-microliter Capitainer^®^ B cards were purchased from Capitainer (Solna, Sweden). The device allows accurate quantitative collection of two 10 µL spots per card. Twenty-microliter Mitra devices were acquired from Neoteryx (Torrance, CA, USA). The device offers volumetric absorptive microsampling of blood (twenty microliters per device). Whatman protein saver cards, each containing five collection spots, were obtained from Sigma-Aldrich (St. Louis, MI, USA).

### 2.3. Blood Sample Collection and Handling

**Figure 1:**
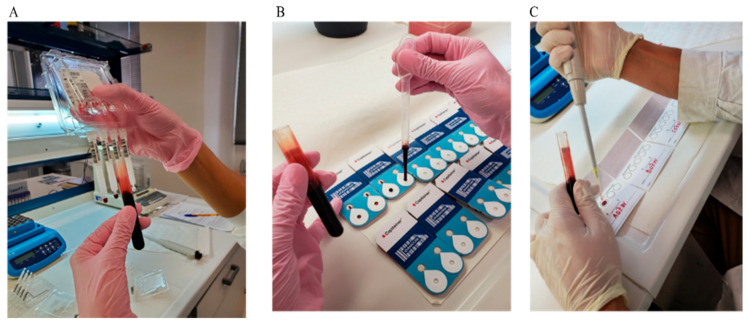
Sampling of BµS devices used in the study. (**A**) Dipping a 20 μL capacity Mitra tip into blood sample, (**B**) pipetting a big drop of blood on a Capitainer device (2 × 10 μL) using a Pasteur pipette, and (**C**) pipetting 20 µL onto a spot on a Whatman card.

Blood samples were collected as part of a pilot study from ten healthy volunteers (five men and five women) following written informed consent to participate. The study was approved by the Research Ethics Committee of the Aristotle University of Thessaloniki (#306272/2022). Samples were collected in the morning after overnight fasting. Two 6 mL samples from an antecubital vein were drawn from each individual in EDTA tubes. A fraction of these was used to obtain plasma after centrifugation. In parallel, BµS was performed as follows: (i) For Capitainer, a drop of blood from venipuncture was placed onto each one of two inlet spots using a Pasteur pipette (each port samples accurately 10 µL). (ii) For Mitra devices, BµS was collected by attaching the tip of the devices to the surface of the collected venipuncture blood for approximately six seconds. (iii) Lastly, five 20 µL aliquots were pipetted onto the five spots of a Whatman card (see Figure 1).

A pooled blood sample was also prepared by combining 500 μL from each volunteer and treated in the same way as the individual samples (that is, plasma preparation and BμS) for protocol optimization purposes. All samples were left to dry at room temperature for 3 h and were then stored at −80 °C in desiccant-containing pouches until analysis.

### 2.4. Analytical Sample Preparation

#### 2.4.1. BµS Extraction Optimization

Four different extraction solvents, that is, MeOH, AcN, MeOH-H₂O 60:40 (*v*/*v*), and AcN-MeOH 70:30 (*v*/*v*), were evaluated. Two Capitainer discs (each 10 µL), one Mitra tip, or one Whatman spot prepared with pooled blood were placed in 1.5 mL Εppendorf tubes and hydrated using 20 µL of H₂O. Then, 300 µL of either extraction solvent was added. Each tube was briefly vortexed, agitated for 5 min, and treated in an ultrasonic bath for 15 min. The BµS device was removed using tweezers, and the tubes were centrifuged (6720× *g* for 10 min at 4 °C). The supernatants (290 µL) were transferred to new tubes, evaporated using a speed vacuum concentrator (Εppendorf Concentrator plus, Stevenage, UK), and reconstituted in 330 µL of H₂O-MeOH 95:5 (*v*/*v*) containing 5 µg/mL of each internal standards, ^13^C-Phe, OC-d3, and BC-d3. The extracts were briefly vortexed and centrifuged as above. The supernatants were then injected into the LC-MS/MS system. The extraction was performed in triplicate per BμS device and solvent.

Procedural blanks were prepared in all tested extraction conditions. Plain devices were placed in Eppendorf tubes hydrated with 20 μL of water and extracted with 350 µL of each of the four different solvents using the same procedure described above.

#### 2.4.2. BµS and Plasma Comparison

Samples from the 10 individuals collected using the three ΒμS devices were extracted using the procedure described above with 350 µL of the optimum extraction solvent, MeOH-H_2_O 60:40 (*v*/*v*). The corresponding plasma samples were extracted using an optimized in-house protocol as follows: 350 μL of AcN-MeOH 70:30 (*v*/*v*) was added to 20 μL of plasma. The samples were vortexed briefly and centrifuged as described above. Then, 290 μL of supernatant was evaporated to dryness as described above and reconstituted in 330 μL of H_2_O-MeOH 95:5 (*v*/*v*) containing the same internal standards previously described. Each extract was divided into three technical replicates, which were then injected into the LC-MS/MS system.

Procedural blanks were prepared for all three devices and plasma. Plain devices were extracted with MeOH-H₂O (60:40) as described in Section 2.4.1. For the plasma procedural blank, 20 μL of water was added to an Eppendorf tube and extracted with 350 μL of AcN-MeOH 70:30 (*v*/*v*) using the procedure described in the paragraph above.

### 2.5. LC/MS-MS Analysis

A Bruker Elute ultra-high-performance liquid chromatograph, coupled to a trapped ion mobility spectrometry time-of-flight MS (Bruker, Billerica, MA, USA) was used. Reverse phase separation was performed using an Acquity UPLC HSS-T3 (C18), 1.8 µm particle size, 2.1 × 100 mm column (Waters, Milford, MA, USA). The aqueous mobile phase was 0.1% formic acid in H₂O (solvent A) and the organic one was 0.1% formic acid in methanol (solvent B). The mobile phase gradient was as follows (percentage of solvent B): 0% from 0 to 1.5 min, linearly increased to 10% from 1.5 to 4 min, linearly increased to 40% from 4 to 8 min, linearly increased to 100% from 8 to 12 min, and held at 100% from 12 to 14 min. Three minutes of equilibration in the initial conditions followed. The flow rate was kept at 0.35 mL/min. The injection volumes were 10 µL for the extraction optimization study and 20 µL for the BµS device and plasma comparison study.

The qTOFMS was operated in data-dependent acquisition mode with a 40-to-800 *m*/*z* scanning mass range at a scanning rate of 5 Hz. The electrospray ionization (ESI) source was operated in positive ion mode with the following parameters: end plate offset 500 V, capillary voltage 4200 V, nebulizer gas pressure 2.2 bars, drying gas flow rate 10 L/min, temperature 220 °C, and quadrupole energy 4.0 eV. The collision-induced decay energy was set for each mass range. Sodium formate solution (10 mM) was used for calibration by direct infusion into the MS at a 10 µL/h flow rate for the first 0.5 min of every injection.

For quality assurance, the samples were analyzed in a randomized order in both the extraction optimization and ΒμS-plasma comparison studies. Additionally, in both studies, quality control (QC) samples were prepared by combining 50 μL of extracts from all samples [31,32]. For the ΒμS-plasma comparison, the QC sample was divided into aliquots and injected at the beginning of the run for system conditioning (6 times) and within the run every 10 samples (12 times).

### 2.6. Data Analysis

The raw MS data files were converted to mzML using Proteowizard (version 3.0.23129). These data are available in the MetaboLights public database [24] under the identifier MTBLS10585. The data were then processed and analyzed using XCMS (version 4.3) in R (version 4.3.0) and MS-DIAL (version 5.2.240424.3-net472). For the extraction optimization study, each device’s data were processed separately. In XCMS, for chromatographic peak picking, the centWave method was used (ppm = 50, peakwidth = c(10, 20), snthresh = 5, intergrate = 2). Refinement to remove overlapping peaks and other artifacts was conducted using the “mergeNeighboringPeaksParam” method (expandRt = 10, expandMz = 0.01, ppm = 10, minProp = 0.75). Alignment was based on the QC samples; therefore, an initial correspondence step, using the “peakDensityParam” method (minFraction = 5/6, binSize = 0.01, ppm = 10, bw = 4), was run to define features in these samples. These features were subsequently used with the “peakGroupsParam” method (minFraction = 0.9, extraPeaks = 50, span = 0.5, subsetAdjust = “average”) to align the whole dataset. A second alignment step was run, using a number of manually selected peaks to target the area between 150 and 400 s this time. The same “peakGroupParam” method was run, except that this time, a table of these “anchor” peaks with their respective *m*/*z* and retention time area in the “peakGroupsMatrix =” parameter was added. A correspondence step was then performed on the entire aligned dataset, setting parameters for the “peakDensityParam” method to minFraction = 0.5, binSize = 0.01, ppm = 10, and bw = 2. The code used for the analysis is available in Github.

For metabolite identification, an in-house library was constructed by analyzing a pooled blood sample (from the same study) using MS-DIAL (version 5.2.240424.3-net472). Possible annotations were matched against multiple public libraries, including MassBank, MassBank-EU, ESI(+)-MS/MS from authentic standards (16,481 unique compounds), and ESI(+)-MS/MS from standards+bio+in silico (16,995 unique compounds), available at https://systemsomicslab.github.io/compms/msdial/main.html#MSP (accessed on 21 May 2024);. Global Natural Products Social Molecular Networking libraries were also used (https://external.gnps2.org/gnpslibrary, accessed on 21 May 2024). The results from the annotation using the in-house library are provided in the Table S10–S13.

In the extraction optimization study, the area was normalized using median scaling to correct the in-between sample variation. The use of IS for normalization was not adopted as its performance was worse than in the median scaling approach. Correction by IS did not improve the precision of QCs for the majority of the features, as could be expected due to the diversity of metabolites. In the BµS-plasma comparison study, the raw data were reproducible, with seemingly no intensity variation throughout the run, and were therefore not normalized. In all cases, features present in procedural blank samples with an average intensity higher than half the average intensity in the experimental samples were flagged as possible contaminants and were subsequently removed from the dataset.

### 2.7. Statistical Analysis

The precision of the QC sample data from the XCMS output was analyzed in MS EXCEL (version 2405) to determine the coefficient variation of the features. Principal component analysis (PCA) and supervised orthogonal partial least-square discriminant analysis (OPLS-DA) were performed in Simca (version 14.1). Univariate data analysis was performed in R (version 4.4.1) on log2-transformed feature abundances. *p* values were adjusted for multiple hypothesis testing using the method of Benjamini and Hochberg [6,33].

## 3. Results and Discussion

### 3.1. Extraction Optimization

The overarching aim of the present study was to evaluate the utility of novel blood microsampling techniques in untargeted metabolomic research. The metabolic fingerprints acquired from three ΒμS devices were compared with those obtained from plasma.

As a first step, we aimed to determine the optimal extraction protocol of metabolites from ΒμS devices. To this end, two pure solvents, MeOH and AcN, and two mixtures, MeOH-H₂O 60:40 (*v*/*v*) and AcN-MeOH 70:30 (*v*/*v*), were tested. The selection of these solvents was based on prior research conducted by our group on the optimal extraction solvent for liquid blood after evaluating various compositions. Additionally, we consulted previous publications that reported the effectiveness of mixtures of water with organic solvents [20,22,34,35]. To evaluate the optimal extraction, a set of criteria was considered: the total number of features detected, the average number of features detected per extraction ± SD, the percentage of missing values among these features, the number of unique features, and the overall feature intensity.

**Figure 2:**
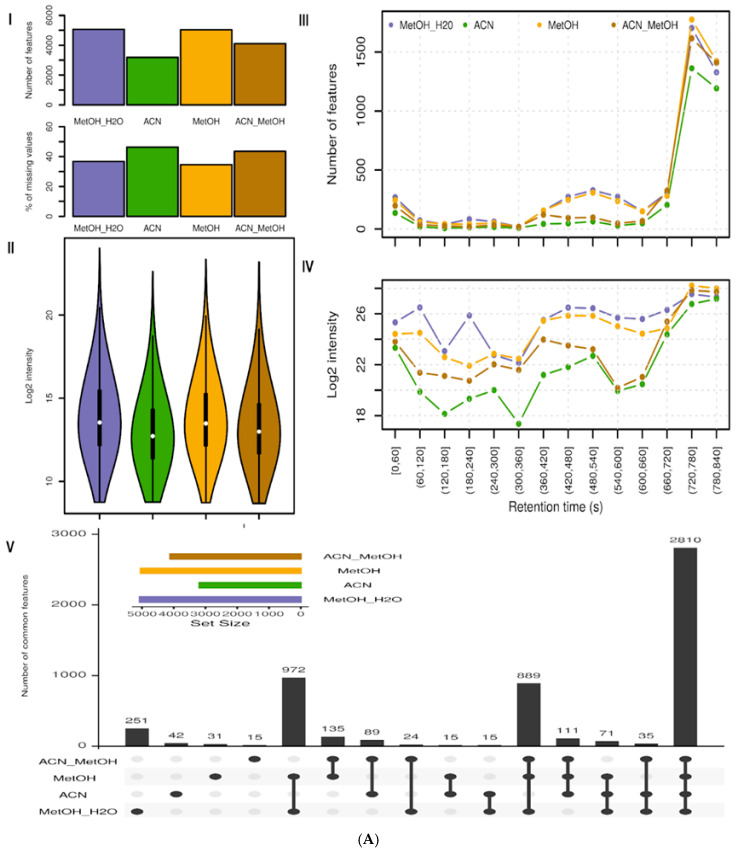

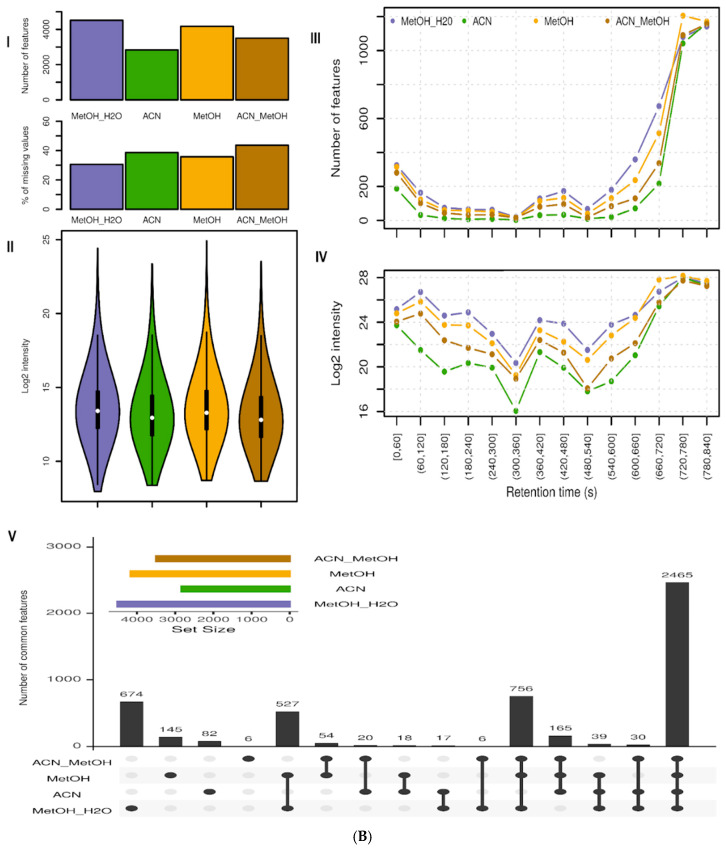

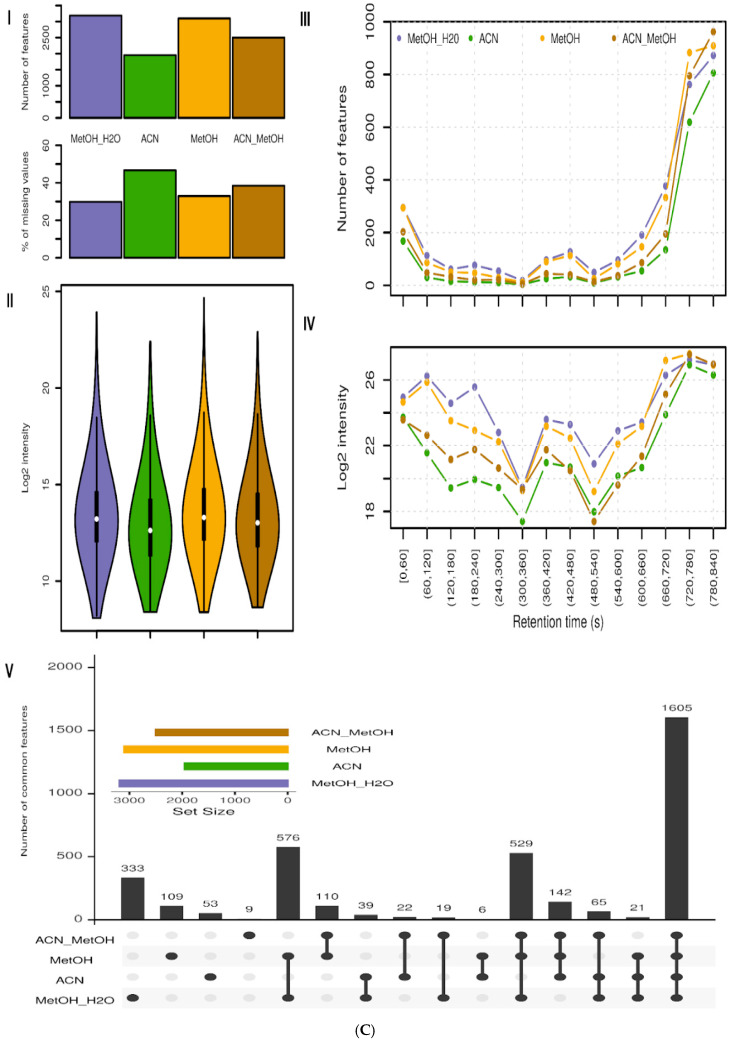
Results of solvent extraction efficiency evaluation for Capitainer (**A**), Mitra (**B**), and Whatman (**C**). (I) Bar chart showing the number of features for each extraction solvent (top) and % of missing features (bottom). (II) Violin plot showing log2 feature intensity distribution for each extraction solvent. (III) Line plot showing number of features at different retention time ranges for each extraction solvent. (IV) Line plot showing log2 of the sum of intensity of features at different retention time ranges for each extraction solvent. (V) Upset plot displaying the unique and common features of each solvent. The first four columns show the number of unique features of the solvent group. The following six columns show the number of features present in two of the compared extracts. The remaining columns represent the features that are common in more than 3 of the extracts analyzed.

After raw data extraction and processing, a matrix of *m*/*z*—retention time features, along with their intensities, were obtained for each BµS device. Only features with a valid signal in two out of three replicates were considered. The highest number of features was detected using MeOH-H₂O 60:40 (*v*/*v*) in all BµS devices (5067 in Capitainer, 4514 in Mitra, and 3187 in Whatman), followed by MeOH (5034 in Capitainer, 4169 in Mitra, and 3098 in Whatman), AcN-MeOH 70:30 (*v*/*v*) (4108 in Capitainer, 3502 in Mitra, and 2501 in Whatman), and AcN (3188 in Capitainer, 2836 in Mitra, and 1953 in Whatman). Very similar findings can be seen by the average number of features in the triplicates, where MeOH-H₂O 60:40 (*v*/*v*) had a higher average number of features, with a similar standard deviation as the other tested solvents, for both Mitra and Whatman. For Capitainer, the results between MeOH:-H₂O 60:40 (*v*/*v*) and pure MeOH were very similar to each other. These results can be seen in Tables S1–S3. A graphical illustration of the number of features detected can be seen in the upper part of Figure 2A(I) for Capitainer, Figure 2B(I) for Mitra, and Figure 2C(I) for Whatman.

In addition, MeOH-H₂O 60:40 (*v*/*v*) yielded the lowest percentage of missing values for both Mitra and Whatman (30.6% and 29.8%, respectively). This represents the number of missing features divided by the total number of features in the replicates, and the results can be seen in bar charts of Figure 2A(I) (for Capitainer), in Figure 2B(I) (for Mitra), and in Figure 2C(I) (for Whatman). The percentages of missing values with MeOH were 35.8% for Mitra and 32.9% for Whatman; with AcN, 38.6% for Mitra and 38.4% for Whatman; and with AcN-MeOH 70:30 (*v*/*v*), 43.7% for Mitra and 46.7% for Whatman. For Capitainer, MeOH was the solvent with the lowest percentage of missing values (36.6%), followed closely by MeOH-H₂O 60:40 (*v*/*v*) (36.7%), whilst AcN-MeOH 70:30 (*v*/*v*) (43.5%) and AcN (46.4%) had the highest percentages of missing values. This criterion reflects the amount of the identified features that were not present in every replicate and, as such, can be used as an indicator of the reproducibility of feature detection per solvent. Thus, it can be concluded that MeOH-H₂O 60:40 (*v*/*v*) was the solvent with the most reproducible results, particularly for Mitra and Whatman. MeOH provided slightly more reproducible results for Capitainer compared with MeOH-H₂O 60:40 (*v*/*v*).

A comparison of the distribution of feature abundances and their averages per extract and device can be seen in Figure 2A(II)–C(II). The log2 signal distributions were similar for all extracts, with MeOH-H₂O 60:40 (*v*/*v*) and MeOH yielding slightly higher abundances on average. When evaluating the signal, i.e., the number of features and their summed intensities along the retention axis, MeOH-H₂O 60:40 (*v*/*v*) and MeOH outperformed the other extraction solvents (see Figure 2A(III,IV)–C(III,IV)). In the latter part of the LC analysis, beginning at 11 min, MeOH performed slightly better than MeOH-H20 60:40 (*v*/*v*), most likely because of the more efficient extraction of the less hydrophilic metabolites, such as fatty acids and acylcarnitines, which are eluted by pure MeOH.

The overlap of the detected features in the different extracts was then examined. In the lower inset (V) of Figure 2, the numbers of intersecting (common) and unique features with the four extraction solvents are shown. Higher overlaps were found between all MeOH-containing solvents. Among all solvents, MeOH-H₂O 60:40 (*v*/*v*) had the highest number of unique features for all microsampling devices, suggesting that it might provide additional information independently of the device used.

Lastly, the log2 sum intensities of the annotated compounds for each extraction solvent in all of the BµS devices were compared as can be seen in Figure S1. Based on this, MeOH-H₂O 60:40 (*v*/*v*) was shown to have the highest sum intensity for the annotated metabolites. In addition, among the annotated metabolites, MeOH-H₂O 60:40 (*v*/*v*) gave the highest peak area for most of the annotations (18 annotations were higher with MeOH-H₂O 60:40 (*v*/*v*) in Capitainer, 39 in Mitra, and 19 in Whatman), followed by MeOH (5 in Capitainer, 2 in Mitra, and 16 in Whatman), AcN-MeOH 70:30 (*v*/*v*) (17 in Capitainer, 1 in Mitra, and 2 in Whatman), and, finally, AcN (0 in Capitainer, 2 in Mitra, and 4 in Whatman). Heatmaps of the annotated metabolites showing the intensities of the annotated metabolites can be seen in Figure S2A–C.

To summarize, MeOH-H₂O 60:40 (*v*/*v*) was found to provide the most efficient extraction conditions for every BµS device considering the number and abundances of the detected as well as annotated features. Thus, it was chosen as the solvent for the extraction of BµS in subsequent experiments. MeOH was a close second, displaying good results for different devices. In contrast, AcN provided the poorest results across all evaluated criteria.

### 3.2. BµS and Plasma Comparison

In a second step, a set of 10 samples on the three BµS and the corresponding plasmas were extracted using the above-defined optimal extraction solvent (MeOH-H₂O) 60:40 (*v*/*v*). For this analysis, a feature was considered when a similar chromatographic peak was identified in at least 50% of each extract’s replicates. The acquired metabolic profiles were compared using specific criteria, which included the total number of features detected per extract, the average number per extract, the number of features with the highest intensity, the number of unique features, % of missing features, the device with the largest number of annotated metabolites with higher intensity, and the precision of the annotated metabolites.

#### 3.2.1. Evaluation Based on Detected Features

Based on the initial quality assessment of the data, it was found that from the three repeated measurements of each sample, one was not acceptable due to a lower signal in comparison to the other two (see Figure S3), most probably due to insufficient sample volume in the vials, and, consequently, they were excluded from further data analysis. Thus, data from only two of the three technical replicates were considered. Similarly, based on QC sample data, from the 12 QCs, 6 were excluded.

There were a total of 8378 features detected in all samples after the removal of contaminants from the blank. The mean Pearson correlation coefficient (r) of all features between the six QCs was 0.942. The median precision of features expressed as a percentage coefficient of variation (%CV) in the QCs was 28.5% CV, with a lower quartile of 19.6% CV and an upper quartile of 41.49% CV. The precision of the features was found to be <30% for 4204 of the total 8378 detected features. The internal standards mean %CVs based on their peak areas in all of the samples were also calculated, showing for PHE-c13 24.01%, for BC-d3 14.12%, and for OC-d3 21.75%. All of the above quality control assessment results can be found in the Tables S3–S9.

**Figure 3:**
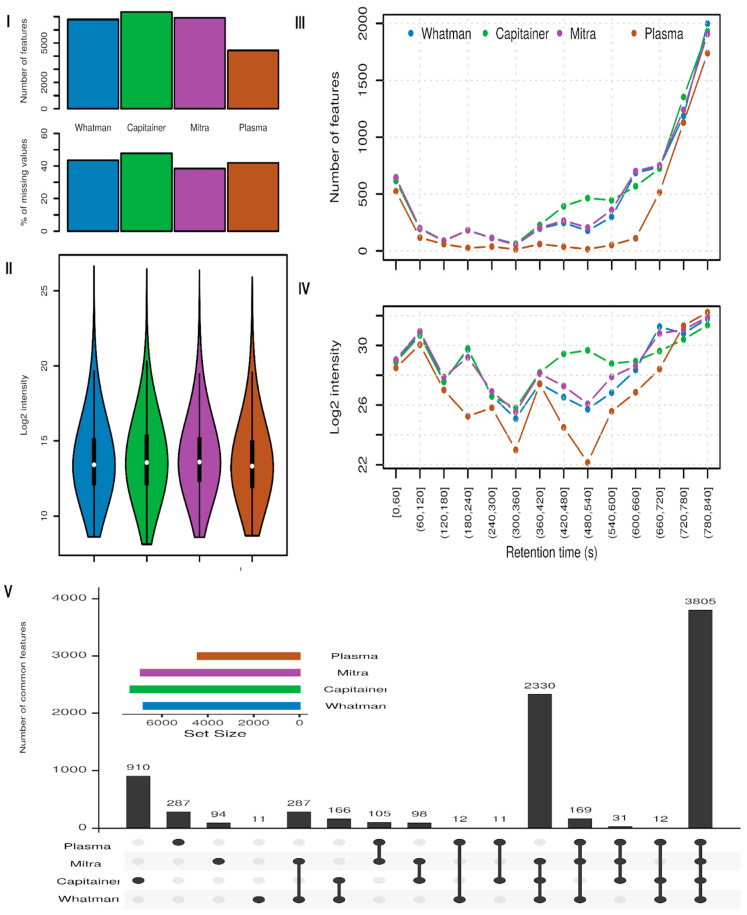
Detected features analysis in BµS and plasma extracts. (**I**) Bar chart showing number of features (top) and % of missing values (bottom). (**II**) Violin plot showing log2 of feature intensity distribution for each BµS and plasma. (**III**) Line plot showing number of features against rt. (**IV**) Line plot showing log2 of the sum of intensity of features against rt. (**V**) Upset plot showing common and unique features. The first four columns show the number of unique features. The following six columns show the number of features present in two of the compared extracts. The remaining columns represent the features that are common in more than 3 of the extracts analyzed.

The highest number of detected features were observed in Capitainer (7403), followed by Mitra (6945), Whatman (6846), and plasma (4455) (see Figure 3(I)). Based on the average number of features per extract, a similar trend can be observed with Capitainer, having, in all individual samples, a higher number of features (see Table S2). Capitainer, on the other hand, had the largest percentage of missing features (47.13%), followed by Whatman (42.76%), plasma (41.73%), and Mitra (38.44%). Considering that the studied samples originate from healthy individuals and no variation in the samples is expected, we could assume that this inconsistency in detection is material-dependent.

The distribution of the intensities of the aforementioned detected features is at a similar level in all four extracts, as shown in Figure 3(II). However, when looking at the distribution of the features and their log2 summed intensities along the retention time axis (Figure 3(III,IV)), a device-specific pattern is observed. Indeed, Capitainer showed improved performance both in terms of the number of features detected and their respective intensity in the 7 to 10 min range, where metabolites of increased lipophilicity are expected to be eluted. Mitra and Whatman performed in similar ways, and plasma seems to consistently have lower amounts of features detected and intensities all along the retention time axis.

This distribution of the signal along the retention time axis was further investigated by looking at one randomly picked representative sample and examining all of its extracts (Figure S4). A large number of features were detected in the ΒμS extracts in the first part of the chromatogram, especially with an *m*/*z* above 200. This is in contrast to the pattern obtained in plasma, which was poorer. Mitra and Whatman showed features spread along the *m*/*z* and rt range and in a similar pattern, while Capitainer had more features with high *m*/*z* after the second half of the chromatogram. A point that should be nevertheless noted here is that the solvent used for the extraction of plasma was the optimum according to prior studies of our group (mixture of AcN-MeOH, used also by other researchers in blood metabolomics studies [34,35]) and not the same used for the dried ΒμS. However, as the major aim of the study was to compare our established protocol in plasma against BµS, this is something that should not be over-considered. In addition, it should be considered that whole blood would be expected to provide a richer profile compared to plasma due to the metabolic content of blood cells. Thus, differences in the profiles of plasma extracts can be explained to some extent.

From the total number of detected features, 3805 were common to all four sample extracts, and 2330 were found in all three ΒμS extracts. In addition, there were also some features specific for each extract: 910 for Capitainer, 94 for Mitra, 11 for Whatman, and 287 for plasma (see Figure 3(V)). This shows an increased detectability of features in the Capitainer device extract compared to the others.

Overall, both Mitra and Capitainer devices provided a higher number of detected features, with Mitra showing higher reproducibility (lower % missing values). On the other hand, Whatman cards showed less satisfactory results regarding the same criteria. Compared to plasma, the BμS seemed to outweigh the obtained profiles, as higher numbers of features and intensities were obtained, thus indicating, by assumption, richer metabolic profiles.

Such findings have been reported in previous studies, where differential metabolite detection specific to the matrix used was observed when Whatman DBS and plasma sample extracts were compared [18]. In that study, a number of metabolites were detected in Whatman DBS but not in plasma. Similar findings have also been reported by Volani et al. [20] while comparing VAMs with plasma extracts. Both qualitative and quantitative differences have been observed in metabolites of plasma and venous blood collected in VAMs. They reported 76 and 79 features (in positive and negative mode) uniquely detected in venous blood, whereas only 13 and 24 (pos and neg) features were unique in plasma. These reports support our findings on the alternative and increased metabolic information acquired by the BμS approaches.

#### 3.2.2. Evaluation Based on Annotated Metabolites

From the full set of detected features, 46 could be annotated using an in-house library built from whole blood analysis from the same study participants. Twenty-three were level one (matched *m*/*z* and rt to standards) and 23 were level two identifications (matched ms/ms to public databases). The mean precision of the annotated metabolites in the QCs was 21.12% CV, which was similar to one of the internal standards used, PHE-c13; BC-d3 and OC-d3 precisions were 14.12% CV and 24.1% CV, respectively. In addition, data from three randomly selected subjects were analyzed for evaluation of the precision of the four extracts. The mean precisions of the identified metabolites were 22.1%, 12.1%, 20.3%, and 12.3% CV in the Capitainer, Mitra, Whatman, and plasma extracts, respectively. All four extracts showed high precision in the annotated metabolites.

**Figure 4:**
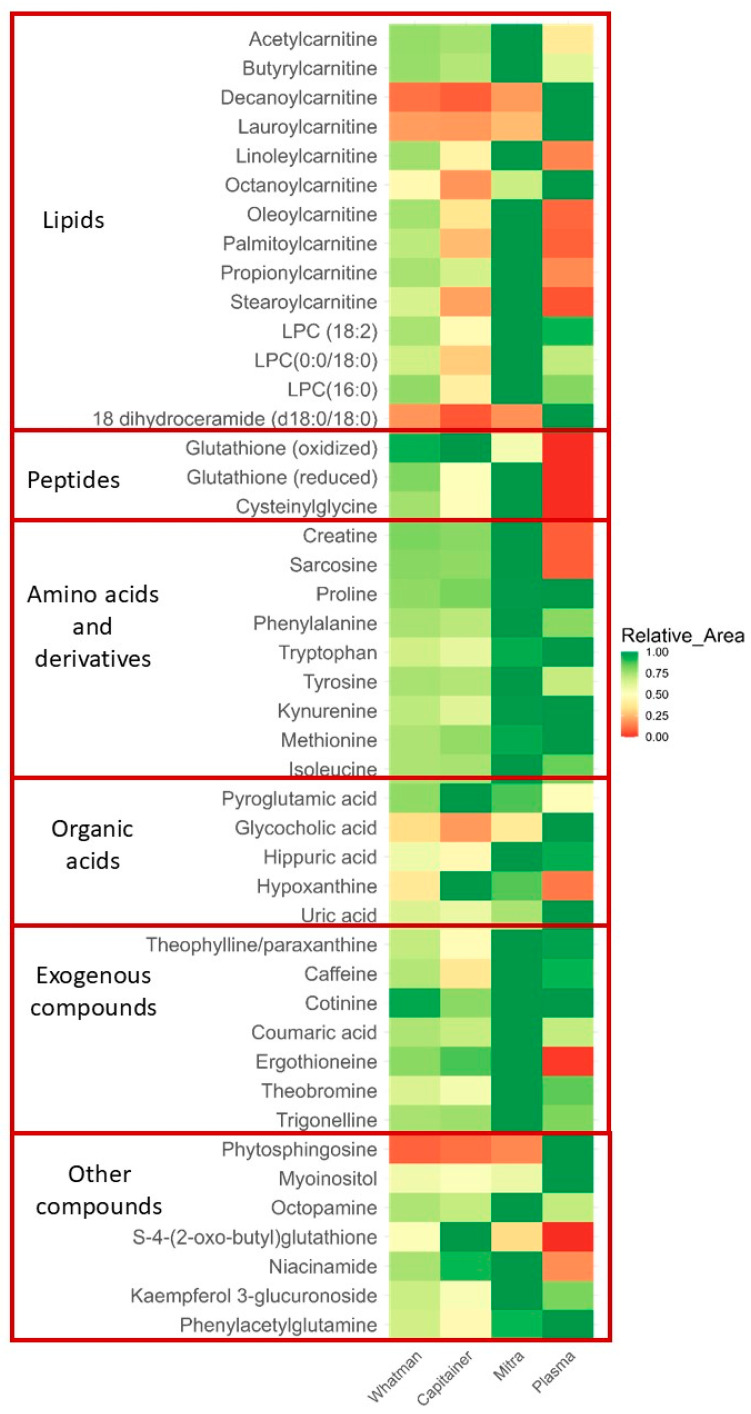
The average intensity of each metabolite in each extract. Color key: red to green = lowest to highest peak area.

From the annotated features, 31 had the highest intensities recorded in the Mitra extracts and 4 in the Capitainer extracts, whereas none of the metabolites showed the highest signal in Whatman (see Figure 4). The 28 metabolites in the Mitra extracts comprised amino acids and derivatives (sarcosine, proline, methionine, isoleucine, tyrosine, phenylalanine, ergothioneine, creatine, and kynurenine), peptides (glutathione reduced and cysteinylglycine), carnitines (acetylcarnitine, propionylcarnitine, butyrylcarnitine, linoleoylcarnitine, palmitoylcarnitine, oleoylcarnitine, and stearoylcarnitine), lipids (lysophosphatidylcholine 18:2, lysophosphatidylcholine(16:0), lysophosphatidylcholine(0:0/18:0), and 1-18:1 lysophosphatidylcholine), a pyridine (nicotinamide), organic acids (coumaric acid and hippuric acid), and also some food intake-related metabolites (caffeine, trigonelline, theobromine, theophylline, p-octopamine, and kaempferol 3 glucuronide).

The four metabolites that were most abundant in the Capitainer extracts were hypoxanthine, glutathione oxidized, s-4-(2-oxo-butyl) glutathione, and pyroglutamic acid. Regarding the plasma extracts, 11 metabolites had higher intensity compared to the BμS extracts. These included carnitines species (octanoylcarnitine, decanoylcarnitine, and lauroylcarnitine), fatty acids and lipids (c18 dihydroceramide (d18:0/18:0), phytosphingosine, and glycocholic acid), an organic acid (uric acid), amino acids and derivatives (tryptophan and phenylacetylglutamine), a pyridine (cotinine), and a sugar (myoinositol).

Analysis of variance (ANOVA) revealed statistically different intensities (*p* < 0.05) between the four extracts for 38 of the 46 metabolites. The eight metabolites that were not statistically different in the extracts mainly included food biomarkers, trigonelline, theobromine, theophylline, and caffeine. Three amino acids and their derivatives, proline, tyrosine, and hippuric acid, and glycocholic acid, a lipid derivative, were also not statistically different in the extracts.

Another important observation was that several important biomarkers, such as ergothioneine, glutathione reduced, glutathione oxidized, s-4-(2-oxo-butyl)glutathione, and their metabolite cysteinylglycine, were not detected in plasma extracts but in the corresponding BμS [36]. The highest mean intensity for glutathione reduced was observed in Mitra and glutathione oxidized in Capitainer. Thus, our results show improved stability for important key metabolites.

Despite the overall low number of annotated metabolites, our results suggest that BµS devices, particularly Mitra, can provide equal or, in some cases, even better coverage and information on blood metabolites. However, it seems that this is highly dependent on the metabolites.

#### 3.2.3. Multivariate Analysis

**Figure 5:**
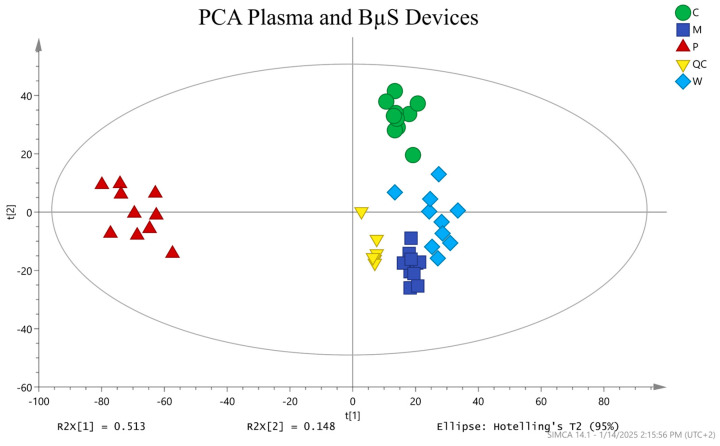
PCA scores plot showing all samples analyzed together with the QCs clustered (R2X [1] = 0.513, R2X [2] = 0.148, Q2 cum [1] = 0.394, and Q2 cum [2] = 0.245). BµS was clearly separated from plasma on PC1. BµS was clustered along PC2, with Whatman and Mitra being slightly similar. QC samples were tightly clustered (excluding the first injection) showing our assay’s high precision. Color key: green = C (Capitainer B), purple = M (Mitra), brown = P (plasma), blue = W (Whatman), and yellow = QC.

For a general evaluation and comparison of the dried blood and plasma-derived metabolic profiles of the 10 samples, PCA was performed. Features from blank samples greater than 50% of the signal in regular samples and QC replicates with >30% CV were then excluded. The average peak area (abundance) of each feature (two replicates per sample) was calculated and log2 transformed. Additionally, features with missing values were excluded from the multivariate analysis. It should be noted that we also imputed the missing values for these features using the k-nearest neighbour (KNN) method and verified that no significant changes were observed in the multivariate analysis (see Figure S5). A clear separation of the plasma samples from all BµS samples can be seen on principal component one (PC1), with the data from the individual BµS clusters along PC2 (Figure 5) with Whatman and Mitra being slightly more similar to each other. A similar observation was noted when examining the raw MS data (Figure S6).

**Figure 6:**
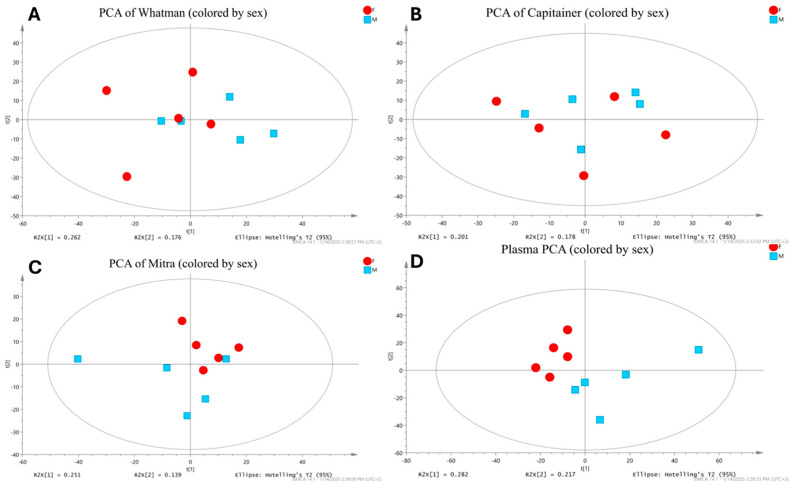
PCA models of metabolome classification based on sex in the four extracts. (**A**) PCA in Whatman did not show separation. (**B**) PCA in Capitainer did not show separation. (**C**) PCA in Mitra did not show separation. (**D**) PCA in plasma shows clusters by sex on PC1. Color key: red = females and blue = males.

When the ability of the sample matrices to discriminate between male and female study participants was examined, an interesting finding was obtained. As an unsupervised approach, separate PCA models using Pareto (par) scaling were performed for every device or plasma extract projecting male against female subject samples (see Figure 6). In that analysis, only plasma samples exhibited a relatively clear separation along the first principal component (PC1). Additionally, Mitra showed a slight tendency of separating male and female participants within the second principal component (PC2). Next, supervised orthogonal partial least-squares discriminant analysis (OPLS-DA) using par scaling was performed for all of the extracts. However, none of the extracts provided valid OPLS-DA models that were able to differentiate males from females. Lastly, in a univariate analysis comparing feature abundances between male and female participants, no feature had a significant difference after adjusting for multiple hypothesis testing. The degrees of freedom, with five replicates per group, were, for the present datasets, given their variance, not large enough to detect the presumably small differences. While not yielding significant results, the plasma dataset seemed to best capture sex differences. Thus, despite the fact that BμS captured rich metabolomic information, it could not significantly differentiate the sex-specific metabolic profiles.

## 4. Limitations and Further Aspects

Based on our results, important conclusions can be drawn regarding the potential use of BμS in untargeted metabolomics. However, further studies are needed for a clearer understanding.

Several aspects of the devices should be considered in future untargeted BμS metabolomics studies, including the make of the device and ease of use. For example, visual inspection after extraction revealed that Mitra tips were more discolored compared to the other devices, indicating extensive extraction (see Supplementary Materials info Figure S7). This could be attributed to the sample tip composition of a polymeric absorbing material, which is different from the cellulose material used in the other two [16,17,37,38,39,40].

In addition, Mitra tips were easily detached from their plastic handles into test tubes without contact. Whatman blood spots, on the other hand, were cut out from the card using scissors, potentially compromising sample integrity due to inconsistent cutting, insufficient cleaning of the scissors between samples, and accidental human contact with the blood spots. Furthermore, Whatman cut-out blood spots were larger in size than the other devices and tightly fitted in the 2 mL Eppendorf tubes. This may have resulted in insufficient extraction due to minimal space for mixing with the solvents. For Capitainer samples, tweezers were used to scoop out the blood spots, and, in many cases, some material remained on the card. Insufficient cleaning of the tweezers between the samples may compromise results as well.

Another important consideration is that this study evaluated the performance of BµS devices using venous blood. Typically, these devices are designed for the collection of capillary blood, usually from a finger prick or newborn heel. In fact, an exact sample volume was applied to the Whatman card, which is not representative of typical use, as it does not collect predefined volumes like the other devices. This factor was not accounted for in our study. To gain more meaningful insights into the potential of ΒμS compared to plasma profiles for metabolomic studies, venous blood profiles should be compared to capillary dried blood. This comparison will be the focus of our upcoming study.

Furthermore, it should be noted that our samples were analyzed after 6.5 months of storage at −80 °C, which could have impacted the stability of metabolites in the different devices and plasma. Multiple publications on the stability of metabolites in dried BµS have focused on short-term storage (a few days to 1 month), primarily involving Whatman and Mitra BµS devices. Jeremy Drolet et al. 2017 reported that the optimal stability for metabolites in Whatman samples was up to 2 weeks at −20 °C; Volani et al. 2023 reported metabolic deterioration in Mitra samples after 6 h at ambient temperature; and Michopoulos et al. 2011 reported improved stability of metabolites stored at −20 °C for up to a month; [20,22,41]. The longest stability study reported was on Mitra, showing improved stability up to 6 months at −80 °C [36]. No reported studies exist on metabolite stability in the Capitainer device in untargeted analysis. As such, we are not sure how storage time impacted our results.

Finally, complementary analytical methodologies or platforms might help us achieve more information and provide a better understanding of blood profiling in BμS as the metabolome coverage highly depends on the analytical setup and sample preparation techniques. This will be our focus in the near future, analyzing the same samples using GC-MS as well as other techniques, and this will be undertaken by associate institutions participating in the same research project.

## 5. Conclusions

BµS devices, particularly novel microsampling devices such as Mitra and Capitainer, seem to provide equal or even superior information on the metabolic content in comparison to plasma, which is the typical specimen for blood metabolomics studies based on the number and intensity of features, as well as precision and stability for some identified metabolites. Despite the richness of the metabolic profiles of BμS, it did not prove useful in revealing biological differentiation. In contrast, since metabolites from blood cells can also be quantified, these sampling techniques might detect additional biomarkers or different biomarkers from conventional plasma samples and can also provide insights into biological or biochemical pathways within the blood cells. These results suggest that a more in-depth investigation of the acquired information is needed for each specific application as a metabolite-dependent trend is obvious. Furthermore, it was demonstrated that, despite sharing a similar concept for sample collection, distinct BμS devices return different metabolic profile information. We can, therefore, conclude that the choice of a specific device might influence the final results of a study and must be carefully selected.

## Data Availability

For more details on the BµS devices vs. plasma solvent optimization experiments, parameter choices, descriptions, and the full code of the analysis. please see the following GitHub repository https://github.com/philouail/solvent_evaluation, accessed on 31 December 2024. Data from our study are available on the MetaboLights public database; MTBLS10585: LC-MS-based global metabolic profile of alternative blood specimens collected by microsampling (https://www.ebi.ac.uk/metabolights/index, accessed on 31 December 2024).

## Supplementary Materials

The following supporting information can be downloaded at: https://www.mdpi.com/article/10.3390/metabo15010062/s1, Figure S1: Violin plots showing the log2 sum intensities of annotated metabolites using different extraction solvents in each extract; Figure S2: The average intensity of each metabolite in each extraction solvent in each BµS device; Figure S3: Plots justifying the exclusion of the second injection of each vial throughout the run (based on QC samples); Figure S4: Feature distribution analysis in one randomly selected individual in all extracts; Figure S5: PCA score plot built by inputting missing values using the kNN method; Figure S6: Heatmap showing the similarity of the total ion content (base peak spectrum) of each sample against each other. Figure S7. BµS devices before and after extraction; Tables S1–S13: Supplementary data analysis.
