# Supplementary material for: LC-MS-Based Global Metabolic Profiles of Alternative Blood Specimens Collected by Microsampling
Source: Metabolites. 2025 Jan 16;15(1):62. doi: 10.3390/metabo15010062 (PMC11767270; doi:10.3390/metabo15010062)
Supplement: Supplementary file 1 [file metabolites-15-00062-s001.zip › Supplementary Figures.pdf]

"

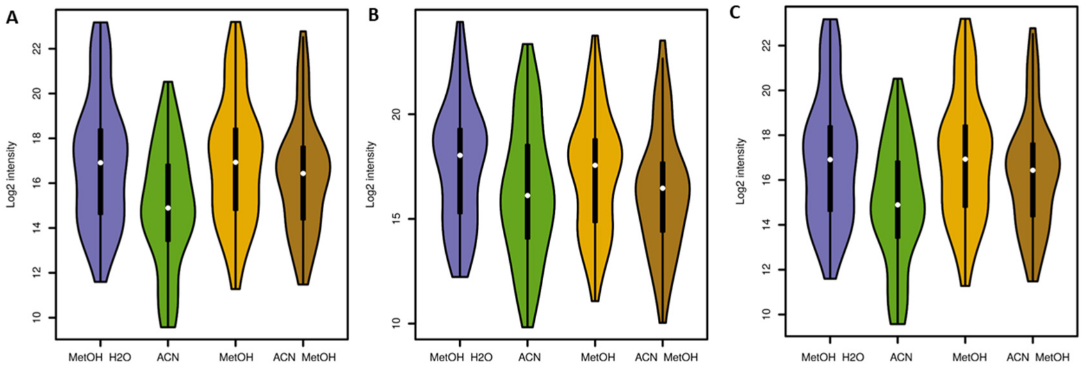

Figure S1. Violin plots showing the log2 sum intensities of annotated metabolites using different extraction solvents in each extract. A) Whatman B) Mitra C) Capitainer. For each extract the intensity was highest using MeOH:H2O (60:40).

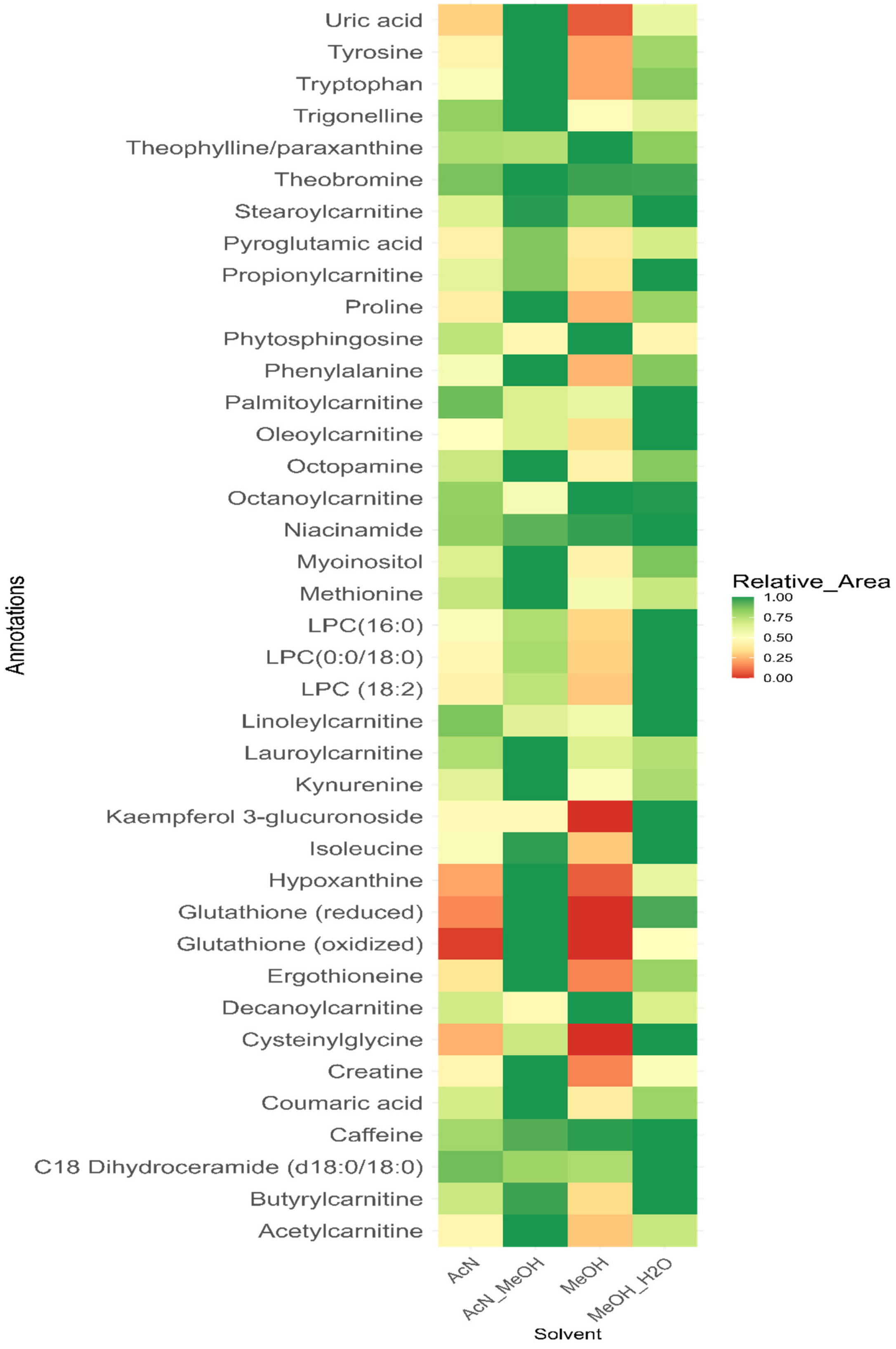

Figure S2A. The average intensity of each metabolite in each extraction solvent using the Capitainer BµS device. **Color key, green to red = lowest to highest peak area.**

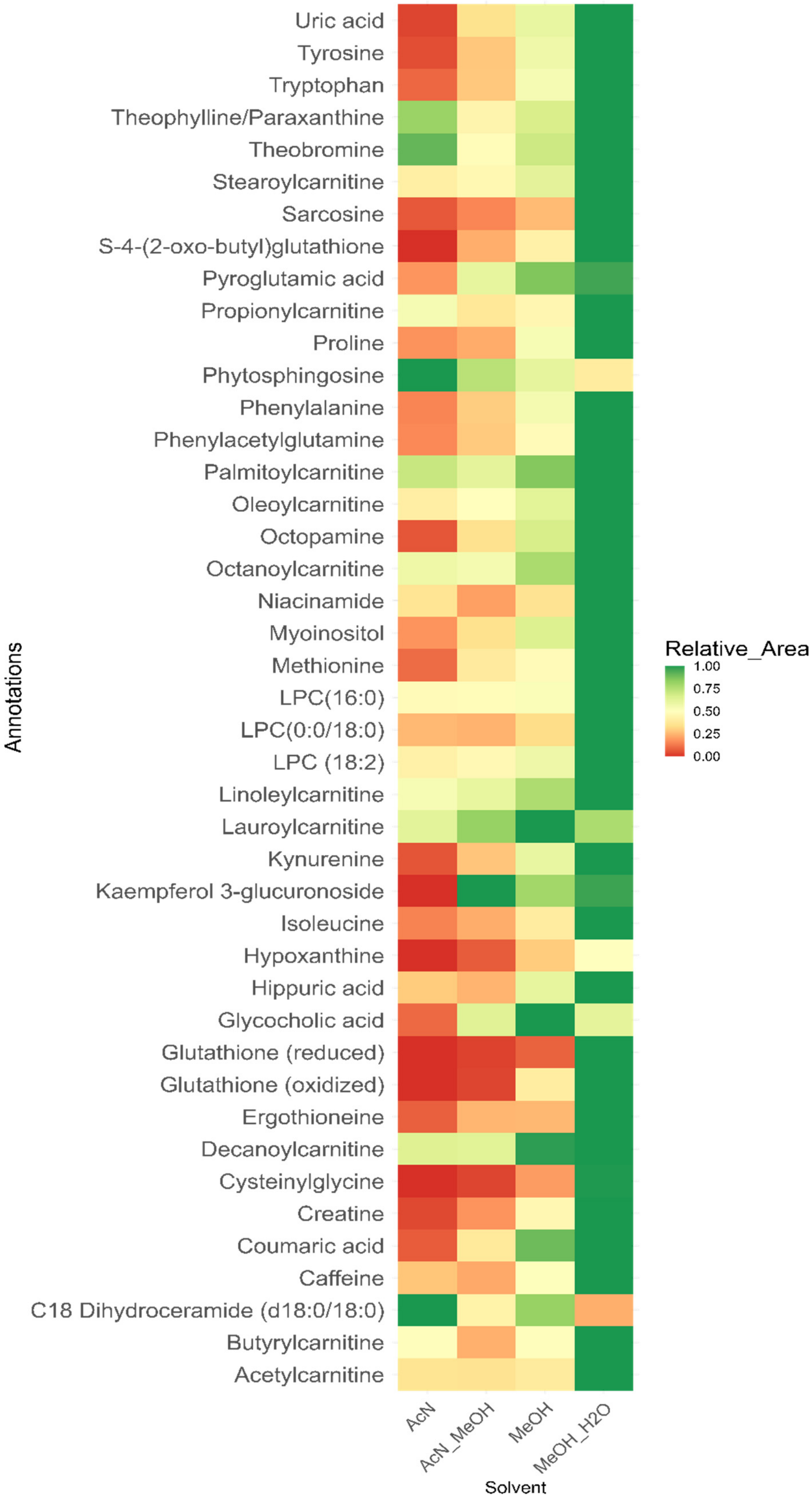

Figure S2B. The average intensity of each metabolite in each extraction solvent using the Mitra BµS device. **Color key, green to red = lowest to highest peak area.**

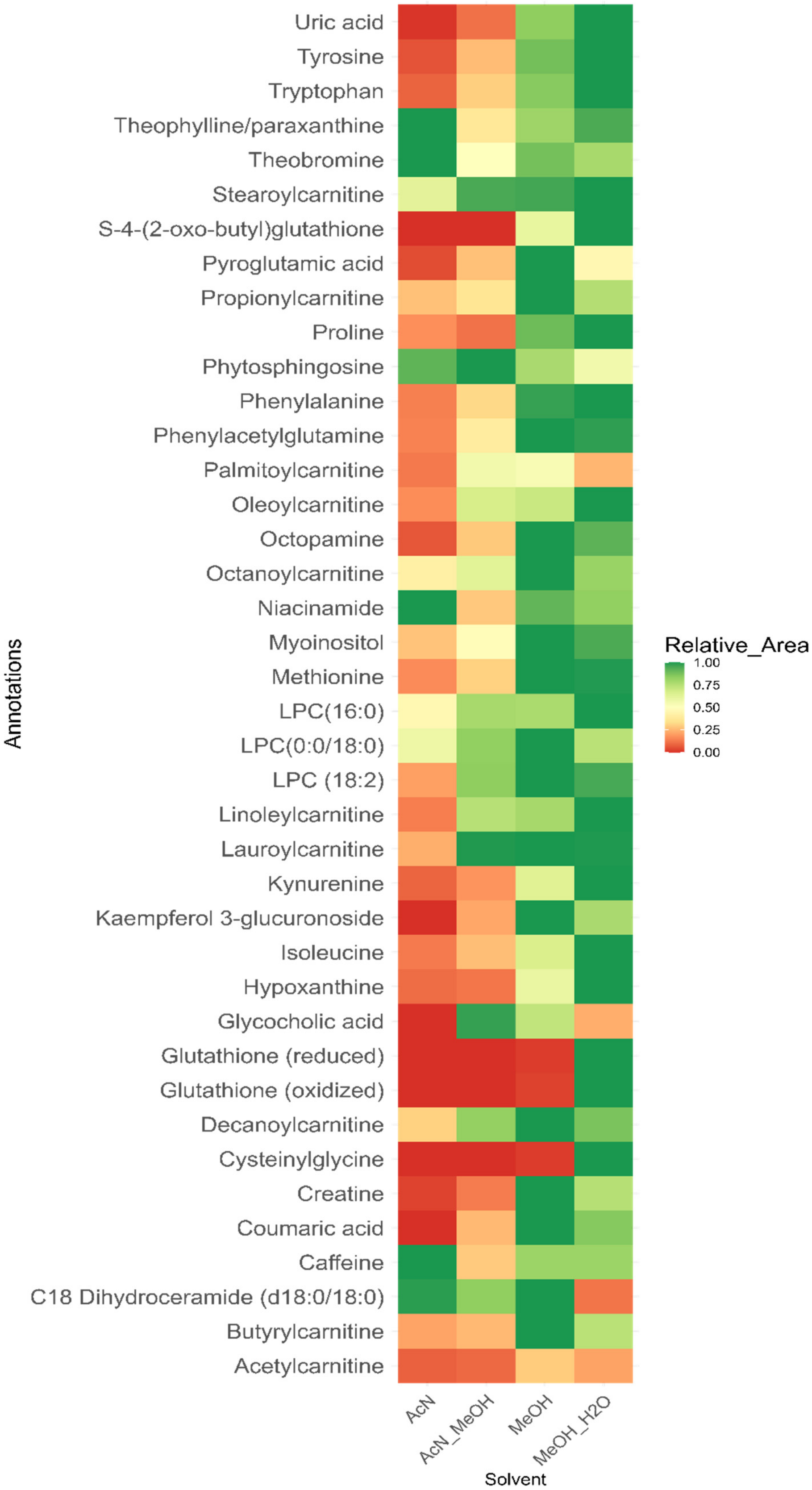

Figure S2C. The average intensity of each metabolite in each extraction solvent using the Whatman BµS device. **Color key, green to red = lowest to highest peak area.**

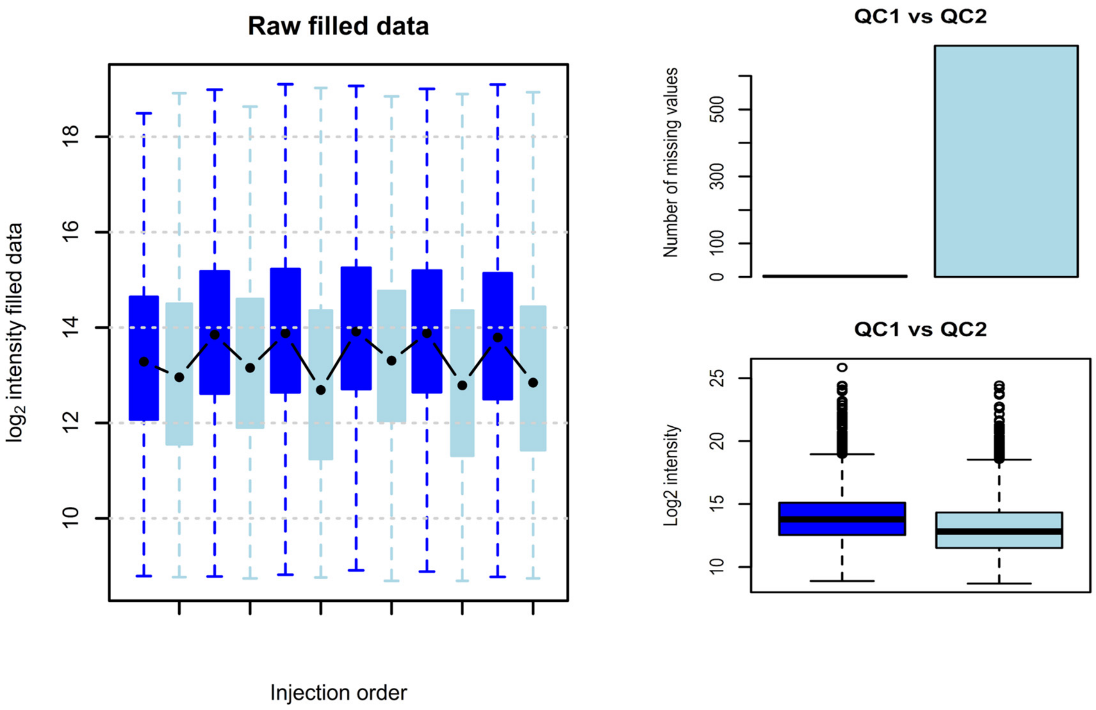

Figure S3: Plots justifying the exclusion of the second injection of each vial throughout the run (based on QC samples). A) Boxplot of log2 sum intensity of features in the first and second QC injections. Every second injection had lower intensity due to lower injection volumes. B) Top bar chart shows the number of missing features in the first and second QC injections. The second QC injections had more missing features. The Bottom boxplot shows the log2 sum intensity of features in the first injection and second injections. **Color key: first injection = dark blue and second injection = light blue.**

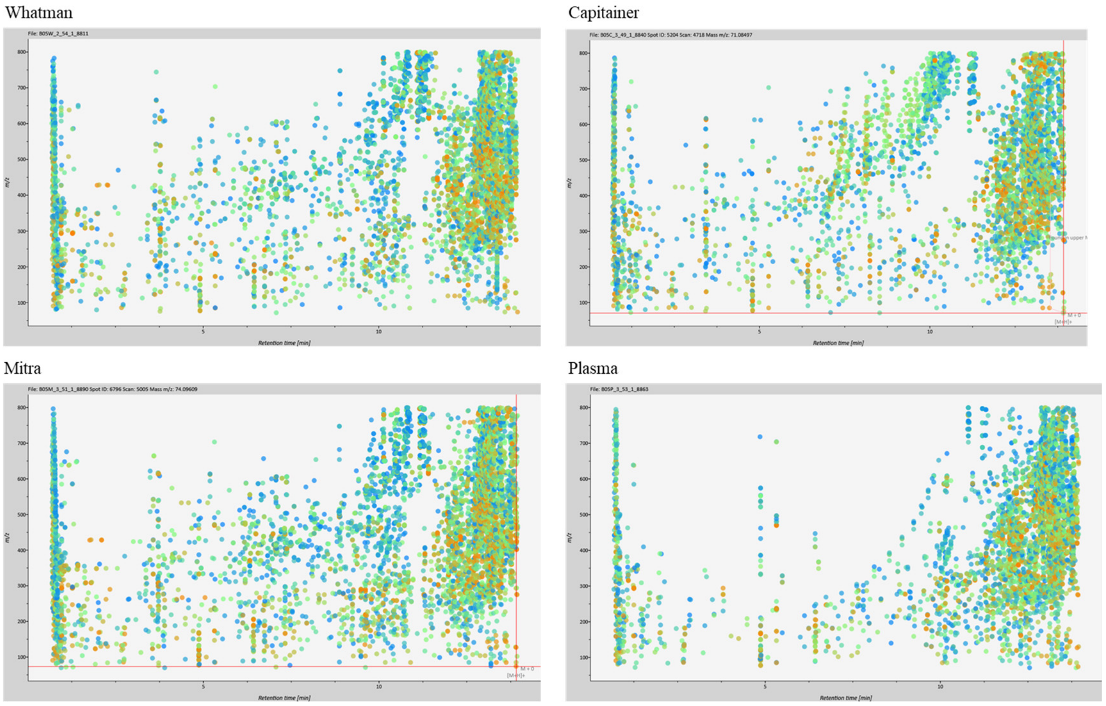

Figure S4: Feature distribution analysis in one randomly selected individual in all extracts. Whatman and Mitra had slightly similar metabolomic patterns compared to plasma and Capitainer. The dried extracts had richer metabolomic information compared to plasma. **Color key: blue to red = lowest to highest intensity.**

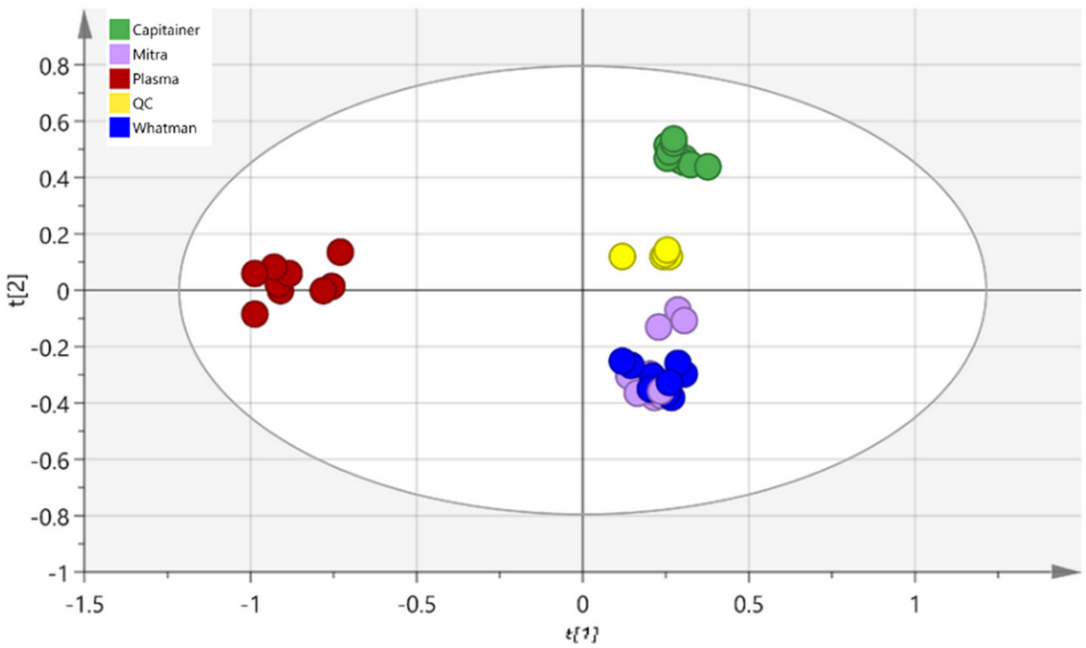

Figure S5. PCA scores plot build by inputting missing values using the kNN method. It is similar to the model built by excluding features with missing values on Fig. 5, the QCs sample clustered together, while plasma and Capitainer seem separated from Whatman and Mitra, which cluster together (R2= 0.749, Q2=0.732).

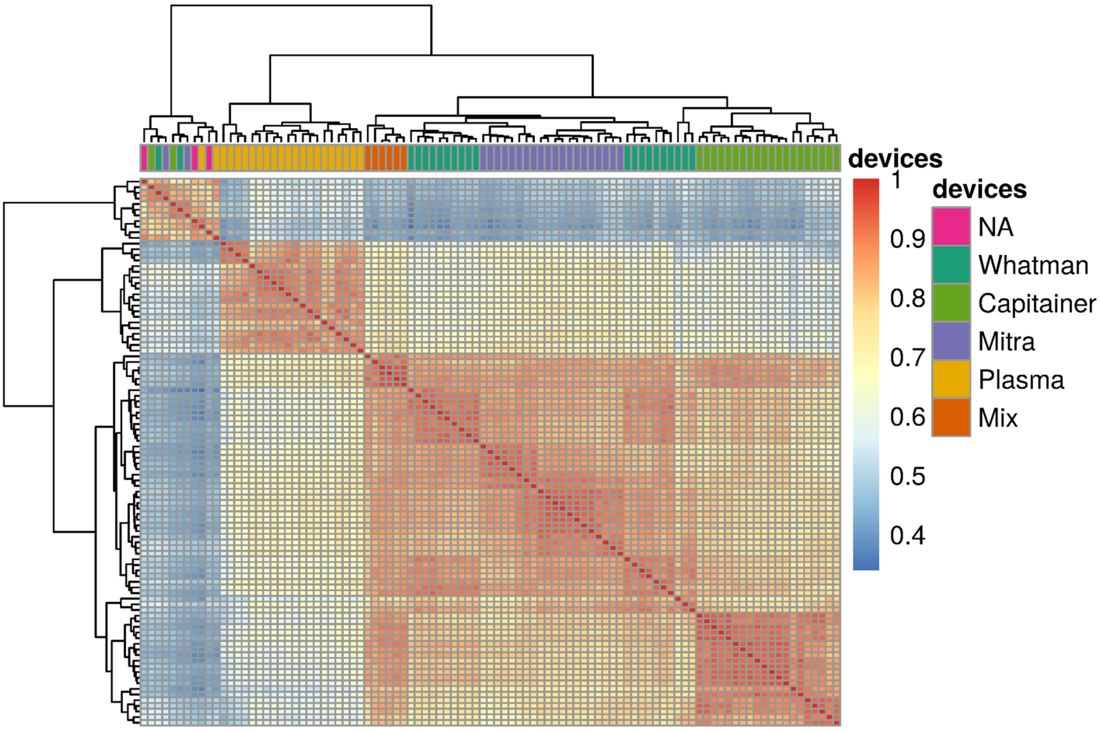

Figure S6. Heatmap showing the similarity of the total ion content (base peak spectrum) of each sample against each other. The ion content of the three BµS devices differed and plasma was different from them. The diagonal cells in red indicated comparison of the same sample. The dendrograms show hierarchical relations between the samples. **Key: similarity increases from 0 to 1 (blue to red); Mix = QC and NA = blank.**

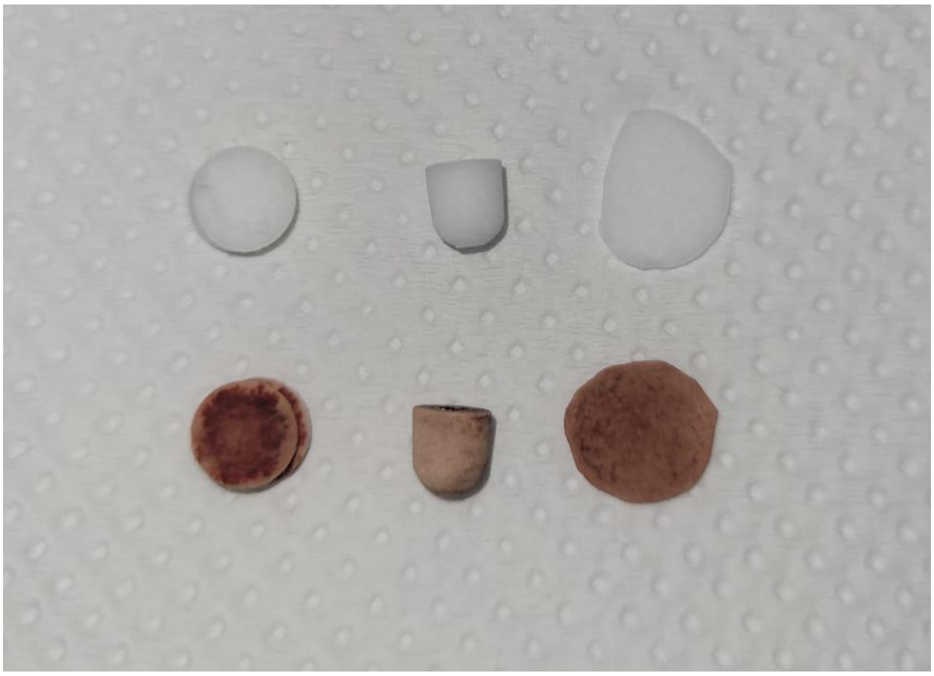

Figure S7. BμS devices before and after extraction. The first row shows unused BμS and the second their appearance after sample extraction. On the second row from left to right: Capitainer (two 10 μL spots), a Mitra tip, and a Whatman blood spot. The Mitra tip was the most faded (discolored) after solvent extraction.

Table S1: Mean and standard deviation of the number of features per extraction solvent for the Mitra device.

Mitra			
	Solvent group	Mean	Standard deviation
MetOH_H2O	MetOH_H2O	3137.67	231.88
MetOH	MetOH	2679.00	98.49
ACN	ACN	1749.33	170.94
ACN_MetOH	ACN_MetOH	1975.00	46.81

Table S2: Mean and standard deviation of the number of features per extraction solvent for the Capitainer device.

Capitainer			
	Solvent group	Mean	Standard deviation
MetOH_H2O	MetOH_H2O	3216.33	293.93
MetOH	MetOH	3297.33	63.96
ACN	ACN	1738.67	197.09
ACN_MetOH	ACN_MetOH	2326.33	290.15

Table S3: Mean and standard deviation of the number of features per extraction solvent for the Whatman device.

Whatman			
	Solvent group	Mean	Standard deviation
MetOH_H2O	MetOH_H2O	2240.33	118.42
MetOH	MetOH	2082.67	147.70
ACN	ACN	1045.67	131.52
ACN_MetOH	ACN_MetOH	1541.67	35.84

Table S4: Mean and standard deviation of the number of features per device in different individuals.

Device	Sample ID	Mean	Standard deviation
Capitainer	01	7280.00	207.89
Capitainer	02	7264.00	326.68
Capitainer	03	6567.50	1300.37
Capitainer	04	6858.50	837.92
Capitainer	05	6667.50	958.13
Capitainer	06	7192.00	246.07
Capitainer	07	7523.50	62.93
Capitainer	08	6679.50	1173.09
Capitainer	09	7170.50	333.05
Capitainer	10	7300.00	103.24
Mitra	01	6934.50	50.20
Mitra	02	6307.00	395.98
Mitra	03	6664.00	418.61
Mitra	04	6919.50	53.03
Mitra	05	6900.50	26.16
Mitra	06	6910.00	43.84
Mitra	07	6862.50	13.44
Mitra	08	6891.00	169.71
Mitra	09	6481.50	726.20
Mitra	10	6923.50	24.75
Plasma	01	4606.00	189.50
Plasma	02	4311.00	32.53
Plasma	03	4563.00	12.73
Plasma	04	4296.50	461.74
Plasma	05	4338.50	12.02
Plasma	06	4528.50	181.73
Plasma	07	4343.00	304.06
Plasma	08	4053.00	148.49
Plasma	09	4269.50	129.40
Plasma	10	4449.50	95.46
Whatman	01	6689.00	272.94
Whatman	02	6742.50	21.92
Whatman	03	6638.00	185.26
Whatman	04	6910.00	162.63
Whatman	05	6309.00	571.34
Whatman	06	6431.50	38.89
Whatman	07	5807.00	905.10
Whatman	08	6681.50	13.44
Whatman	09	6448.50	566.39

Whatman	10	6800.50	77.07
